# NF-κB Activation Is Essential for Cervical Cell Proliferation and Malignant Transformation

**DOI:** 10.3390/ijms26062493

**Published:** 2025-03-11

**Authors:** Hui Chen, Qianwen Cui, Wulin Yang

**Affiliations:** 1 Anhui Province Key Laboratory of Medical Physics and Technology, Institute of Health and Medical Technology, Hefei Institutes of Physical Science, Chinese Academy of Sciences, Hefei 230031, China; ch2027@mail.ustc.edu.cn (H.C.); cqw0501@mail.ustc.edu.cn (Q.C.); 2 Science Island Branch, Graduate School of University of Science and Technology of China, Hefei 230026, China

**Keywords:** NF-κB, cervical intraepithelial neoplasia, cervical squamous cell carcinoma, immunohistochemistry, hyperplasia

## Abstract

NF-κB, a multifunctional transcription factor, is linked to cancer initiation and progression. As a key immune mediator, it may play a crucial role in HPV-induced cervical carcinogenesis. However, consensus is lacking on the activation timing of NF-κB during the transition from cervical intraepithelial neoplasia (CIN) to cervical squamous cell carcinoma (CSCC). In this study, immunohistochemical analysis was performed to examine RELA, one of the important members of the NF-κB family, and phospho-RELA expression in different cervical lesions. Then, we analyzed NF-κB regulation of differentially expressed genes (DEGs) in cervical lesions vs. normal tissues. Gene enrichment identified oncogenic DEGs, followed by expression and survival analyses. The impact of NF-κB activation on cervical cell proliferation, migration, and oncogenic regulation, as well as the effects of inhibiting NF-κB, were examined. Our study showed that NF-κB activation starts in cervical simple hyperplasia and intensifies as CIN evolves to CSCC. NF-κB-regulated DEGs show stage-specific functions: immune regulation in CIN and cancer promotion in CSCC. Short-term NF-κB activation boosts cervical cell proliferation and migration, which is reversible by an NF-κB inhibitor. Long-term NF-κB activation promotes the expression of cancer-promoting genes in normal cells and also maintains them in cancer tissues, which is linked to poorer prognosis. Inhibiting NF-κB downregulates these genes in cancer cells and suppresses the oncogenic abilities of cervical cancer cells. Collectively, NF-κB activation initiates during the simple hyperplasia stage of cervical cells, stimulating proliferation, migration, and oncogene expression. Throughout the transition from CIN to CSCC, NF-κB activation progressively intensifies, and its long-term activation promotes carcinogenesis. Thus, NF-κB is crucial in mediating cervical oncogenic transformation.

## 1. Introduction

According to the 2022 World Health Organization (WHO) statistics, cervical cancer ranks fourth in both incidence and mortality among female malignancies globally, with approximately 660,000 new cases and 350,000 deaths annually [1]. This disease constitutes a major global health challenge [2]. Cervical squamous cell carcinoma (CSCC), an aggressive malignancy arising from the cervical squamous epithelium [3], accounts for approximately 75% of invasive cervical cancers [4]. Following human papillomavirus (HPV) infection, normal cervical epithelium undergoes cervical intraepithelial neoplasia (CIN), progressing through low-grade (CIN1) to high-grade (CIN2-3) lesions before developing into invasive carcinoma [5]. Simple hyperplasia of cervical epithelial cells, a benign proliferative state lacking cellular atypia, typically precedes CIN development.

Clinical studies demonstrate that even with standardized treatment, CIN patients retain a significantly elevated risk of cervical cancer compared to those without CIN history [6]. While current HPV vaccines display certain preventive efficacy [7,8], the therapeutic outcomes for advanced cervical cancer remain unsatisfactory [6]. Currently, the prognosis for cervical cancer treatment is poor [6], necessitating continued in-depth exploration of its underlying mechanisms to provide a theoretical basis for the development of new therapeutic approaches.

The NF-κB family, a ubiquitously expressed family of transcription factors [9], comprises five REL protein members: RELA (p65), RELB, c-REL, NF-κB1 (p105/p50), and NF-κB2 (p100/p52) [10]. The RELA(p65)/p50 heterodimer represents the predominant functional configuration [9]. It has been reported that the NF-κB signaling pathway is constitutively activated in multiple types of tumors, including cervical cancer, and is involved in a series of tumor phenotypes, such as proliferation, invasion, and lymphangiogenesis [11].

Several reports have been published regarding the activation of NF-κB in cervical cancer, yet the specific activation pattern of NF-κB remains controversial [5,12,13,14,15]. Branca et al. [12] reported that NF-κB only exhibits 20–30% nuclear expression in CIN3 and CSCC. Nair et al. [13] demonstrated a high nuclear positivity rate of RELA in high-grade squamous intraepithelial lesions (HSIL) and CSCC cells. Prusty et al. [14] reported that RELA is primarily expressed in the cytoplasm during CIN and CSCC, with almost no nuclear expression. The nuclear positivity rate of RELA (representing the degree of NF-κB activation) in CIN and CSCC cells reported by different studies varies greatly, and it remains unclear when NF-κB is activated during the transformation from cervical intraepithelial neoplasia to cervical cancer.

In this study, we aimed to explore the activation pattern of NF-κB during the development of cervical lesions and its impacts on the proliferation, migration, and oncogenic regulation of cervical cells to provide a new theoretical basis and potential molecular targets for the diagnosis and intervention of cervical cancer.

## 2. Results

### 2.1. NF-κB Is Progressively Activated in Cervical Simple Hyperplasia, CIN, and CSCC

**Figure 1:**
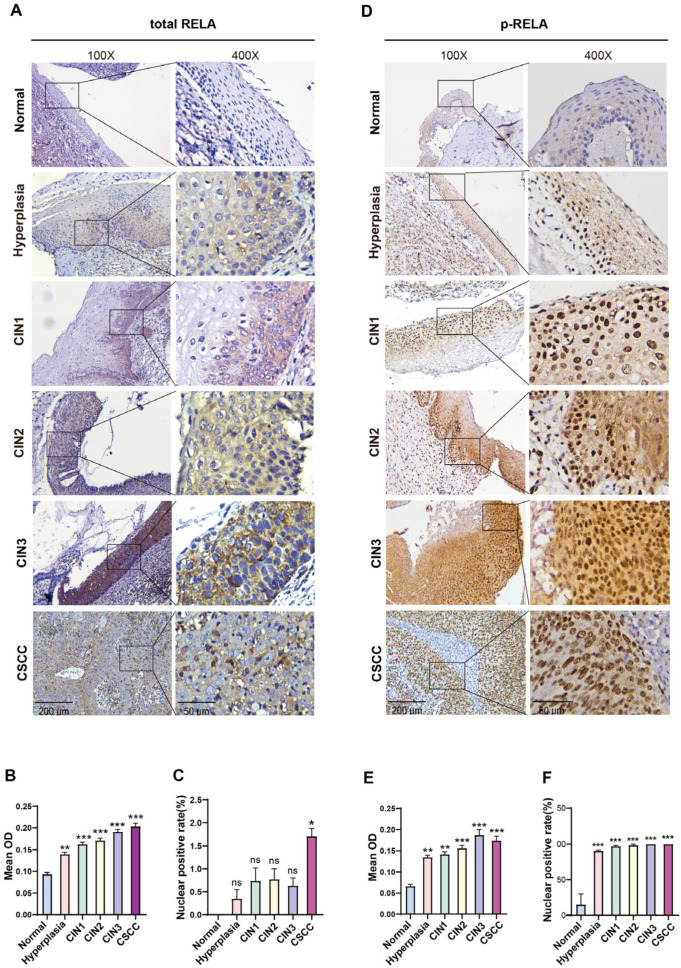
NF-κB is progressively activated in normal, hyperplastic, CIN1, CIN2, CIN3, and CSCC tissues. (**A**) Immunohistochemical analysis of total RELA protein expression in normal, hyperplastic, CIN1, CIN2, CIN3, and CSCC tissues. Scale bars: 200 μm for the left panels (100×) and 50 μm for the right panels (400×). (**B**) Statistical analysis of immunohistochemcal staining intensity (Mean OD) for total RELA in normal, hyperplastic, CIN1-3, and CSCC tissues. (**C**) Statistical analysis of the percentage of nuclear positivity for total RELA in normal, hyperplastic, CIN1-3, and CSCC tissues. (**D**) Immunohistochemical analysis of p-RELA protein expression in normal, hyperplastic, CIN1, CIN2, CIN3, and CSCC tissues. Scale bars: 200 μm for the left panels (100×) and 50 μm for the right panels (400×). (**E**) Statistical analysis of immunohistochemical staining intensity for p-RELA in normal, hyperplastic, CIN1-3, and CSCC tissues. (**F**) Statistical analysis of the percentage of nuclear positivity for p-RELA in normal, hyperplastic, CIN1-3, and CSCC tissues. ns: no significance, * *p* < 0.05, ** *p* < 0.01, *** *p* < 0.001.

To investigate the role of NF-κB in cervical lesion development, immunohistochemical analysis was conducted to examine the expression of the RELA of the NF-κB family in 38 cervical epithelial tissue samples at various pathological stages, including normal samples, simple hyperplasia, CIN1, CIN2, CIN3, and CSCC. The results demonstrated that RELA exhibited weak expression in the cytoplasm of normal cervical cells but was not expressed in the nucleus (Figure 1A). From simple hyperplasia to CIN and CSCC, the cytoplasmic expression of RELA gradually increased (Figure 1A,B). The nuclear expression also progressively intensified throughout the process, from simple hyperplasia to CIN and CSCC, with nuclear positivity rates of 0.35%, 0.74%, 0.78%, 0.63%, and 1.71%, respectively (Figure 1A,C).

To more accurately detect the activation status of NF-κB in cervical tissues at different grades, we assessed the expression of phospho-RELA (ser536). The results indicated that p-RELA was weakly expressed in the cytoplasm of normal cervical cells and only present in a small proportion of nuclei (Figure 1D). Notably, we found that p-RELA expression in the cytoplasm significantly increased from the stage of simple hyperplasia in cervical epithelial cells, accompanied by a gradual increase in nuclear expression throughout the transition from CIN to CSCC (with nuclear positivity rates of 90%, 97%, 99%, 100%, and 100%, respectively) (Figure 1D–F). These findings suggest that NF-κB activation initiates during simple hyperplasia in cervical cells and persists throughout the entire process of transformation from CIN to CSCC.

### 2.2. NF-κB Regulates the Expression of Different Functional Clusters of Genes at Various Stages of Cervical Lesions

**Figure 2:**
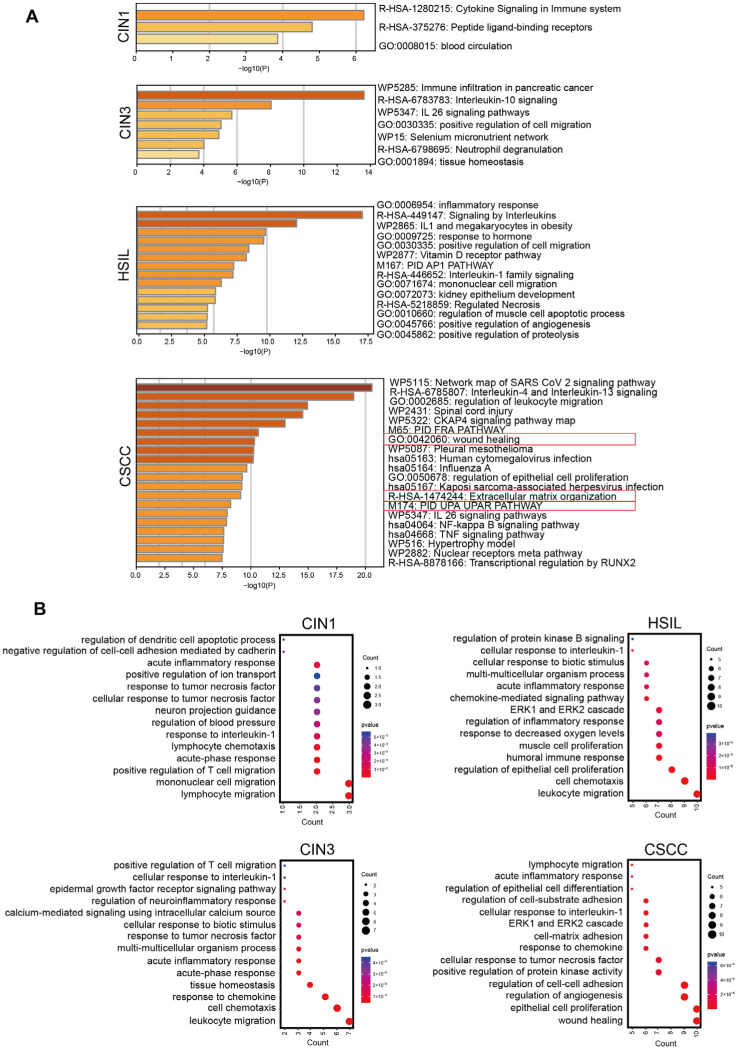
NF-κB regulates the expression of different genes at various stages of cervical lesion progression to cervical cancer. (**A**) Metascape enrichment analysis of DEGs from four groups (CIN1, CIN3, HSIL, CSCC). The red box is used to highlighted the pathways associated with carcinogenesis. (**B**) GO enrichment analysis of DEGs from four groups.

Next, we analyzed gene expression data from cervical tissues in the Gene Expression Omnibus (GEO) database and identified four unique sets of differentially expressed genes—at the CIN1, CIN3, HSIL, and CSCC stages, respectively—in contrast to normal cervical tissues. Subsequently, we used the TRRUST tool to predict the key transcriptional regulators of these four sets of differentially expressed genes (DEGs) and found that all four groups were regulated by RELA [16] (Supplemental Table S1). Specifically, RELA regulated the expression of 8, 16, 40, and 47 DEGs in the CIN1, CIN3, HSIL, and CSCC stages, respectively (Supplemental Table S2). Using the the Metascape tool [17], we conducted an enrichment analysis on the four groups of DEGs regulated by RELA (Figure 2A). The results showed that the DEGs regulated by RELA during cervical intraepithelial neoplasia (CIN1, CIN3, and HSIL) stages were mainly enriched in interleukin-related pathways and inflammatory response pathways, which are associated with immune regulation. Notably, the DEGs at the high-grade intraepithelial neoplasia stages (HSIL and CIN3) were also enriched in pathways positively regulating cell migration, suggesting an increase in cell migration behavior during advanced cervical intraepithelial lesions. Unlike the CIN stages, the DEGs regulated by RELA in the CSCC stage, besides being enriched in interleukin and inflammatory response-related pathways, were also enriched in wound healing, extracellular matrix organization, and PID-UPA-UPAR pathways. These pathways are closely related to tumorigenesis, and include DEGs such as *FN1*, *ICAM1*, *PLAU*, *SERPINE1*, *MMP1*, *MMP12*, *MMP13*, *CXCL1*, and *VEGFA*, which are associated with tumor angiogenesis, migration, and extracellular matrix degradation. The results of Gene Ontology (GO) analysis were consistent with these findings [18] (Figure 2B). These results indicate that as CIN evolves into CSCC, the function of NF-κB also changes, transitioning from an initial role in immune response to a later involvement in tumorigenesis.

### 2.3. Short-Term Activation of NF-κB Promotes Proliferation and Migration of Normal Cervical Cells

**Figure 3:**
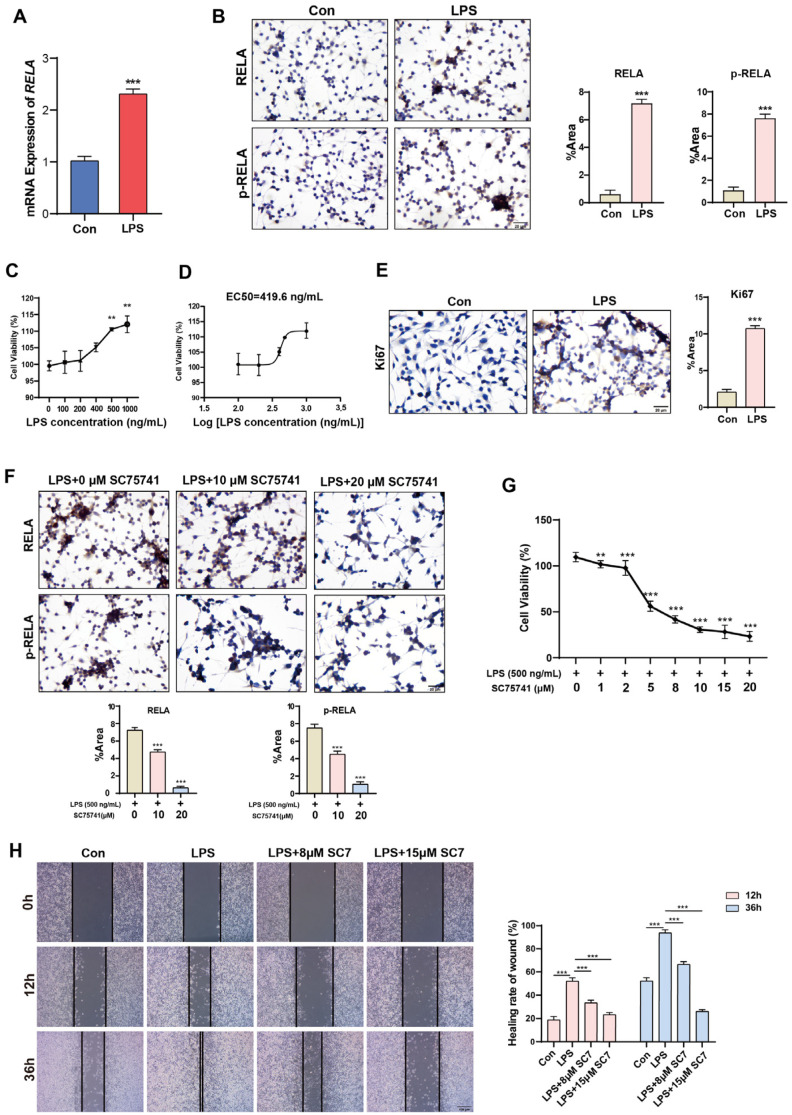
Short-term activation of NF-κB promotes the proliferation and migration of HcerEpic cells. (**A**) RT-qPCR experiment detecting the upregulation of *RELA* mRNA levels in HcerEpic cells treated with LPS (500 ng/mL). (**B**) Immunocytochemical experiment detecting the positive expression levels of RELA and p-RELA in HcerEpic cells stimulated by LPS. ImageJ software was used to quantify the percentage area (%Area) of positive expression areas of RELA and p-RELA. (**C**) CCK-8 assay detecting the effect of different concentrations of LPS on the proliferative ability of HcerEpic cells. (**D**) Calculation of the EC50 curve for HcerEpic cells treated with LPS based on cell viability. (**E**) LPS treatment promotes the expression of Ki67 protein in HcerEpic cells. (**F**) SC75741 treatment inhibits the promoting effect of LPS on the expression of RELA and p-RELA in HcerEpic cells. (**G**) Detection of HcerEpic cell viability under different concentrations of SC75741 and LPS co-treatment. (**H**) Scratch wound healing assay detecting the changes in migration ability of HcerEpic cells after LPS and SC75741 treatment. Scale bar: 20 μm. ** *p* < 0.01, *** *p* < 0.001.

Previous experiments have shown that the activation of NF-κB occurs initially during the stage of simple hyperplasia, leading to the hypothesis that NF-κB activation may be beneficial for the proliferation of cervical epithelial cells. By treating normal cervical epithelial cells HcerEpic with lipopolysaccharide (LPS) for a short duration, it was observed that LPS stimulation significantly increased the mRNA expression of *RELA* (Figure 3A) and upregulated the protein expression of both RELA and p-RELA (Figure 3B). When HcerEpic cells were treated with LPS at different final concentrations (100, 200, 400, 500, 1000 ng/mL), it was found that LPS promoted the proliferation of HcerEpic cells in a dose-dependent manner (Figure 3C), with an EC50 of 419.6 ng/mL (Figure 3D). Therefore, a concentration of 500 ng/mL was primarily used for LPS in subsequent experiments. Consistent with the above findings, LPS stimulation increased the expression of the proliferation factor Ki67 in HcerEpic cells, further indicating its proliferative effect on HcerEpic cells (Figure 3E). In parallel with LPS stimulation, cells were also treated with the NF-κB inhibitor (SC75741). Compared to the control group, the addition of SC75741 significantly reduced the positive expression of RELA and p-RELA proteins in HcerEpic cells in a dose-dependent manner, indicating that SC75741 inhibited the activation of RELA by LPS (Figure 3F). Inhibiting NF-κB activation also significantly attenuated the proliferative effect of LPS on the cells, suggesting that the proliferative effect of LPS on HcerEpic cells is regulated through the activation of NF-κB (Figure 3G). Additionally, we examined the effect of NF-κB activation on the migration ability of HcerEpic cells using a wound healing assay. It was found that LPS promoted the migration of cervical epithelial cells by activating NF-κB; when NF-κB activation was inhibited, the migration ability of the cells was significantly reduced (Figure 3H).

### 2.4. Long-Term Activation of NF-κB Promotes the Expression of Oncogenes

**Figure 4:**
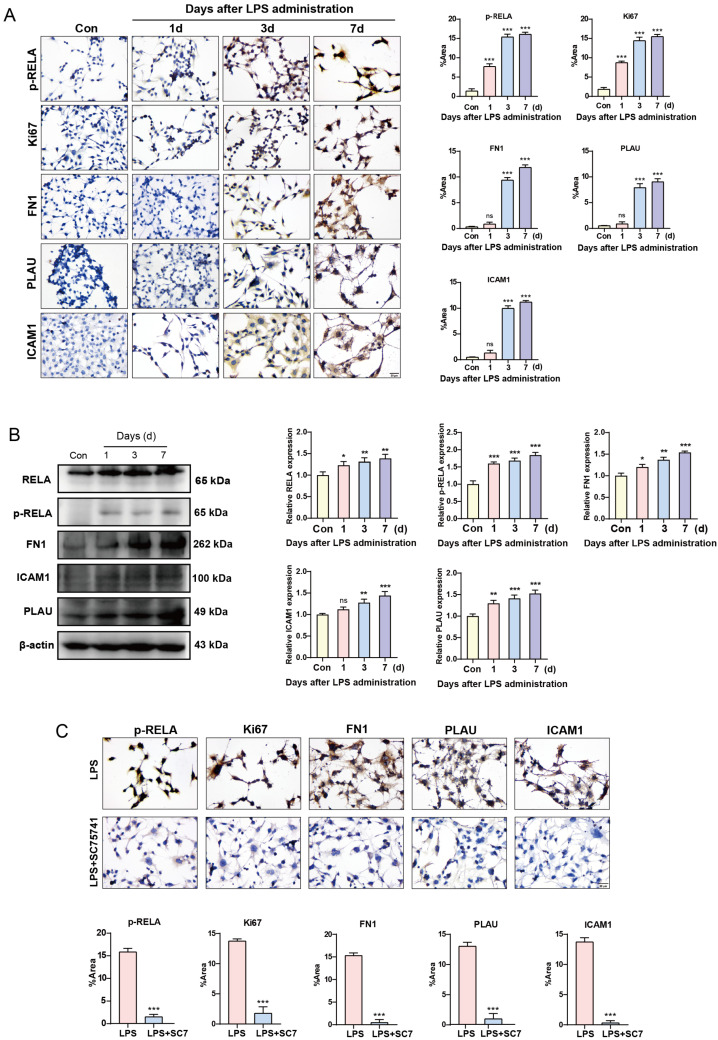
Long-term activation of NF-κB promotes the expression of oncogenes in HcerEpic cells. (**A**) Immunocytochemical experiment detecting the changes in protein expression of p-RELA, Ki67, FN1, PLAU, and ICAM1 in HcerEpic cells after 1, 3, and 7 days of LPS treatment. (**B**) Western blot experiment detecting the changes in protein expression of RELA, p-RELA, FN1, PLAU, and ICAM1 in HcerEpic cells after 1, 3, and 7 days of LPS treatment. (**C**) Immunocytochemical experiment detecting the changes in protein expression of p-RELA, Ki67, FN1, PLAU, and ICAM1 in HcerEpic cells treated with LPS alone and in combination with SC75741. Scale bar: 20 μm. ns: no significance, * *p* < 0.05, ** *p* < 0.01, *** *p* < 0.001.

We treated HcerEpic cells with LPS for 1, 3, and 7 days, respectively, to detect changes in the expression levels of p-RELA, Ki67, and various potential oncogenic DEGs, such as FN1, PLAU, and ICAM1. Compared to the control group, LPS treatment for 1 day significantly promoted the expression of p-RELA and Ki67 but had no obvious effect on the expression of FN1, PLAU, and ICAM1. After 3 days of LPS treatment, there was a significant increase in the expression of FN1, PLAU, and ICAM1. By the 7th day of LPS treatment, the positive expression of p-RELA, Ki67, FN1, PLAU, and ICAM1 was further enhanced (Figure 4A). Western blot results were consistent with these findings (Figure 4B). Cells were treated with a combination of LPS and the NF-κB inhibitor SC75741. Compared to the LPS-only treatment group, the addition of SC75741 significantly inhibited the expression levels of p-RELA, Ki67, FN1, PLAU, and ICAM1, indicating that the increased expression of oncogenic DEGs is regulated through the activation of NF-κB (Figure 4C).

**Figure 5:**
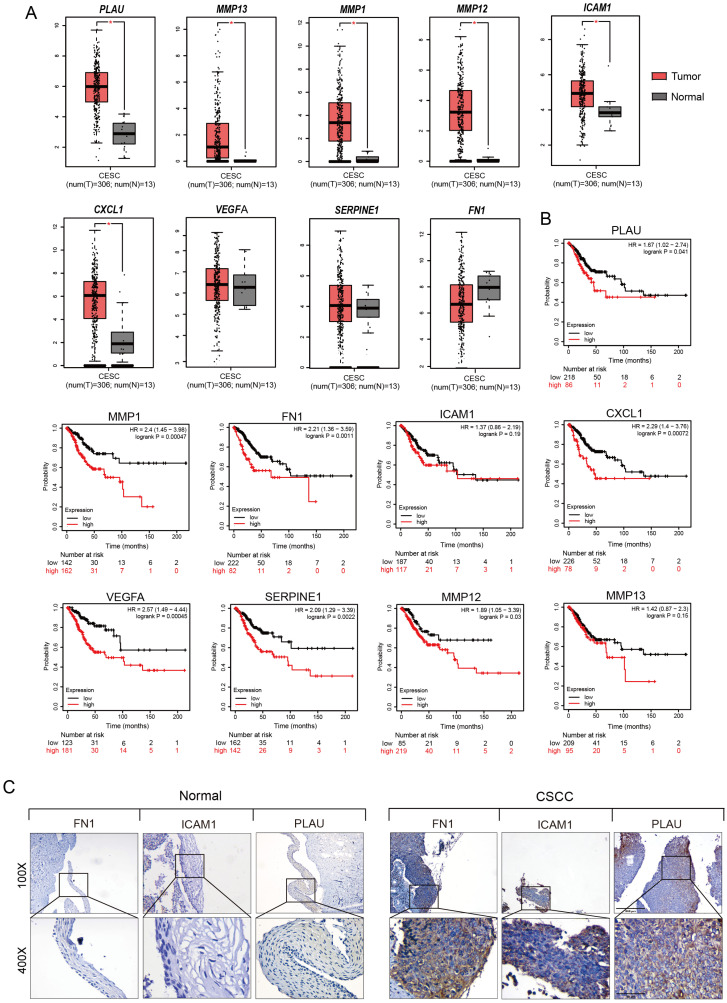
Expression analysis and survival analysis of oncogenic DEGs in normal cervical and cervical cancer tissues. (**A**) GEPIA analysis of mRNA expression levels of 9 oncogenic DEGs in normal cervical and cervical cancer tissues. (**B**) Survival curves of cervical cancer patients with high expression of 9 DEGs and overall survival (OS). (**C**) Immunohistochemical detection of the expression levels of FN1, ICAM1, and PLAU in normal cervical and cervical cancer tissues. Scale bar: In the upper panel (100×), 200 μm; in the lower panel (400×), 50 μm. * *p* < 0.05.

Previous gene enrichment analysis found that RELA regulates the enrichment of potential oncogenic DEGs, such as *FN1*, *ICAM1*, *PLAU*, *MMP1*, *MMP12*, *MMP13*, *SERPINE1*, *CXCL1*, and *VEGFA*, in cancer-related pathways at the CSCC stage. We utilized the GEPIA2 database to analyze the expression levels of these DEGs in cervical cancer and almost normal cervical tissues and found that the mRNA expression of almost all DEGs was higher in cervical cancer tissues than in normal cervical tissues [19] (Figure 5A). Furthermore, compared to normal cervical tissues, the protein expression of FN1, ICAM1, and PLAU was significantly upregulated in cervical squamous cell carcinomas (Figure 5C). Additionally, Kaplan-Meier (KM) survival analysis showed that increased expression of all nine oncogenic DEGs was positively correlated with poor overall survival (OS) [20] (Figure 5B).

### 2.5. NF-κB Regulates Oncogenic Gene Expression in CSCC Cells and Promotes Tumorigenesis

**Figure 6:**
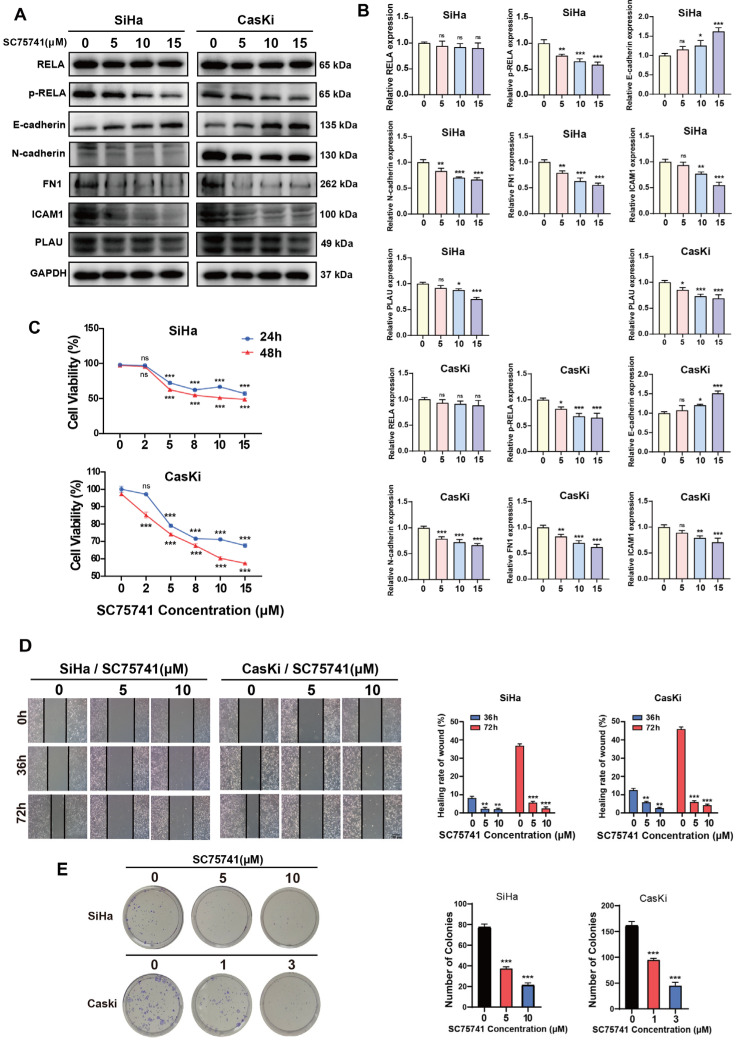
NF-κB regulates the expression of oncogenic DEGs and malignant phenotypes in cervical squamous cancer cells. (**A**,**B**) Western blot detecting the expression of RELA, p-RELA, E-cadherin, N-cadherin, FN1, ICAM1, and PLAU in SiHa and CasKi cells. (**C**) CCK-8 assay detecting the proliferative ability of SiHa and CasKi cells after treatment with different concentrations of SC75741 for 24 and 48 h. (**D**) Representative images of wound healing in SiHa and CasKi cells after scratch injury for 36 and 72 h in control and SC75741 treatment groups. Scale bar: 100 μm. (**E**) Colonies formed by SiHa and CasKi cells in control and SC75741 treatment groups. Data represent three replicates of a representative experiment. ns: no significance, * *p* < 0.05, ** *p* < 0.01, *** *p* < 0.001.

Next, we investigated the regulatory role of NF-κB on the expression of potential oncogenic DEGs, such as *FN1*, *ICAM1*, and *PLAU* in the CSCC cells. Compared to the control group, treatment with SC75741 significantly reduced the protein expression levels of FN1, ICAM1, and PLAU in CSCC cells (SiHa and CasKi), with a dose-dependent effect (Figure 6A,B).

Furthermore, we conducted cellular experiments to examine the relationship between NF-κB and the malignant phenotypes of CSCC cells. After treating the cells with SC75741, we assessed cell proliferation, migration, clonogenicity, and epithelial-mesenchymal transition (EMT) capabilities using CCK-8, wound healing, clonogenic, and western blot assays, respectively. Compared to the control group, the cell proliferation, migration, and clonogenic abilities of the SC75741-treated group were significantly inhibited, with a dose-dependent effect (Figure 6C–E). Western blot results showed that compared to the control group, the SC75741-treated group exhibited decreased N-cadherin protein expression and increased E-cadherin protein expression (Figure 6A,B). These results indicate that inhibiting NF-κB can suppress oncogenic behaviors such as EMT in cervical carcinoma cells.

## 3. Discussion

NF-κB, serving as a pivotal inflammatory regulatory factor, undergoes constitutive activation in numerous types of tumors, constituting a critical link in the inflammation-cancer chain. Our study reveals that NF-κB activation initiates during the stage of simple hyperplasia in the progression of cervical lesions and persists throughout the entire evolution from cervical intraepithelial neoplasia to cervical squamous cell carcinoma. Cytological experiments demonstrate that short-term activation of NF-κB induced by LPS stimulates the proliferation and migration of normal cervical epithelial cells, whereas prolonged NF-κB activation promotes the expression of oncogenes. Therefore, persistent NF-κB activation may represent a significant contributory factor in the development of cervical cancer.

The NF-κB signaling pathway is constitutively activated in various types of tumors, including cervical cancer [13], pancreatic cancer [21], ovarian cancer [22], and others. Previously, several reports have described the activation of RELA in precancerous lesions of the cervix and cervical cancer tissues. Nair [13] and colleagues found that RELA is only expressed in the cytoplasm in normal cervical tissues and low-grade lesions (CIN1-2), but shows nuclear expression in high-grade lesions (CIN3) and invasive cancers (CSCC). Li [5] and colleagues reported that a small fraction of RELA is expressed in the nucleus in normal cervical tissues and CIN1, with increased nuclear expression in HSIL and CSCC. In our study, RELA expression was nearly undetectable in normal cervical cells but demonstrated a marked elevation during the stages of simple hyperplasia, CIN, and CSCC, predominantly localizing to the cytoplasm with only weak nuclear expression. NF-κB typically requires phosphorylation for activation to exert its transcriptional regulatory role, and phosphorylated proteins generally constitute a small fraction of the total protein. This may explain the relatively low nuclear positivity rate for total RELA protein. Using an anti-phospho-RELA (ser536) antibody for detection, we found that phospho-RELA has only weak cytoplasmic expression in normal cervical tissues, but shows significantly enhanced nuclear expression starting from simple hyperplasia of cervical epithelial cells and continues to increase during the transition from CIN to CSCC. This indicates that NF-κB activation may begin at a very early stage of cervical lesions (the clinically considered normal phase of simple hyperplasia of the cervix), rather than at the later stage of cervical lesions—HSIL—as previously thought.

Bioinformatics analysis performed in this study has revealed that NF-κB regulates downstream genes with distinct functions at different stages of cervical lesions. During the CIN stage, the genes regulated by NF-κB are primarily associated with immune regulation, potentially exerting an anti-tumor effect. However, at the CSCC stage, the genes regulated by NF-κB shift towards promoting tumorigenic behaviors such as cell migration and extracellular matrix degradation. It is speculated that prolonged activation of NF-κB may be the reason for its potential oncogenic role in the late stages of cervical lesions. In CSCC, NF-κB can regulate the expression of potential oncogenes such as *FN1*, *ICAM1*, *PLAU*, *MMPs*, and *VEGFA*. The GEPIA2 database indicates that these oncogenes exhibit upregulation at the mRNA level in cervical cancer, except for *FN1*. Nonetheless, there are reports that *FN1* mRNA expression is significantly higher in cervical cancer compared to normal cervical tissue [23]. Notably, immunohistochemistry and western blot assays confirmed that FN1, PLAU, and ICAM1 are significantly upregulated in cervical cancer tissues. VEGF and ICAM1 have been proven to play crucial roles in the metastasis of cervical cancer and are associated with advanced stages and recurrence of the disease [24,25]. Both FN1 and PLAU play pivotal roles in extracellular matrix degradation, facilitating the migration and invasion of cervical cancer cells [23,26].

It is believed that throughout the entire process from HPV infection of cervical cells to the development of cervical cancer, NF-κB activity undergoes continuous shifts between activation and inhibition to maintain persistent viral infection and the transformation of infected cells [9]. HPV infection initiates in the basal cells of the cervix [27]. Generally, upon HPV invasion into host cells, the E6 and E7 oncogenic proteins play crucial carcinogenic roles [28]. The E6 oncoprotein can cause proteasome-dependent degradation of p53, while the E7 oncoprotein blocks the function of retinoblastoma protein (pRb), thereby disrupting the cell cycle, promoting cell proliferation, and simultaneously inhibiting DNA repair and apoptosis, leading to the accumulation of genetic mutations and malignant transformation in infected cells [29,30]. However, most HPV infections are transient and do not necessarily lead to cancer. Therefore, there exist additional synergistic mechanisms. Reports suggest that during early HPV infection, the E1 oncoprotein can induce NF-κB activation and increased NF-κB activity can inhibit HPV viral genome replication and transcription, exerting an antiviral function [31]. Simultaneously, activated NF-κB triggers innate and adaptive immune responses, assisting in viral clearance [9,32]. To counteract this viral replication inhibitory activity, HPV E7 can inhibit NF-κB activity by suppressing the IKK complex, thereby blocking NF-κB nuclear entry and its binding to DNA elements [33,34]. Alternatively, HPV E6 seems to be able to inhibit the transcriptional activity of RELA [35]. Studies have found that with persistent HPV infection, NF-κB activity increases again [9]. Constitutively activated NF-κB regulates the expression of genes related to proliferation, anti-apoptosis, and angiogenesis [36,37], while the originally dominant tumor suppressor gene functions are downregulated [38]. This aligns with the findings of our study. The functionality of NF-κB is complex, as it can exert tumor-suppressive effects by regulating immune processes on the one hand, and regulate the expression of oncogenes on the other. This functional complexity may be governed by tissue-specific immune microenvironments, where NF-κB activation can exhibit either anti-tumor or pro-tumor effects depending on contextual immunological conditions. Mechanistic studies demonstrate that NF-κB activation induces tumor cells to secrete chemokines such as CCL2, which facilitates monocyte recruitment to tumor sites and their subsequent differentiation into tumor-associated macrophages (TAMs) [39,40]. During early phases, TAMs may participate in anti-tumor immunity through pro-inflammatory cytokine and reactive oxygen species production. However, under conditions of sustained NF-κB activation, TAMs predominantly polarize into immunosuppressive M2-type macrophages, thereby promoting tumor immune evasion [39]. During systemic immune responses, NF-κB activation modulates bone marrow hematopoietic stem cells to orchestrate immune cell production and differentiation, thereby expanding immune cell populations [41]. However, aberrant activation of NF-κB induces systemic inflammatory responses. This persistent inflammatory status disrupts the differentiation of hematopoietic stem cells and the generation of immune cells within bone marrow niches, which ultimately compromises immune homeostasis and impairs the host’s tumor surveillance capacity through diminished immune cell-mediated clearance mechanisms [42]. Consequently, NF-κB activation exerts dual regulatory roles in tumor immunity through coordinated modulation of both in situ and systemic immune responses, demonstrating context-dependent anti-tumor or pro-tumor outcomes.

In summary, through immunohistochemical analysis combined with cytological experiments, this study has, for the first time, discovered that NF-κB is activated during the stage of simple hyperplasia of cervical cells and gradually intensifies and persists during the transformation from cervical intraepithelial neoplasia to cervical squamous cell carcinoma. Short-term activation of NF-κB only promotes the proliferation of cervical epithelial cells, whereas prolonged activation leads to the expression of oncogenes and enhances cellular malignancy, serving as a driving factor for the development of cervical cancer. Therefore, NF-κB represents a promising molecular target for the diagnosis and intervention of cervical lesions.

## 4. Materials and Methods

### 4.1. Sample Collection

The 38 paraffin-embedded tissue blocks used in this study were collected from Hefei Cancer Hospital, the affiliated hospital of Hefei Institutes of Physical Science, Chinese Academy of Sciences, Hefei, China. These included 4 samples of normal cervical tissue, 7 samples of basal cell hyperplasia, 5 samples of CIN1, 6 samples of CIN2, 8 samples of CIN3, and 8 samples of CSCC. All samples were used for immunohistochemical experiments.

### 4.2. Analysis of Differentially Expressed Genes

The Gene Expression Omnibus database (http://www.ncbi.nlm.nih.gov/geo/, accessed on 10 March 2023), a public repository containing vast amounts of gene expression data, was utilized to download two microarray datasets (GSE51993 and GSE7803 [43]) for analysis. The GSE51993 dataset contains gene expression data of 7 normal cervical epithelium samples, 9 samples of CIN1, and 8 samples of CIN3. The GSE7803 dataset includes gene expression data of 10 normal cervical epithelium samples, 7 samples of HSIL, and 21 samples of CSCC. We used the GEO2R online tool (http://www.ncbi.nlm.nih.gov/geo/geo2r, accessed on 10 March 2023) to analyze the DEGs in CIN1 vs. normal, CIN3 vs. normal, HSIL vs. normal, and CSCC vs. normal groups, respectively. Subsequently, DEGs from the GSE7803 dataset were screened according to the criteria of |log_2_FC| ≥ 1 and adjusted *p*-value < 0.05, while DEGs from the GSE51993 dataset were screened based on the criteria of |log_2_FC| ≥ 1 and *p*-value < 0.05. A total of 107, 167, 654, and 905 DEGs were identified in the CIN1, CIN3, HSIL, and CSCC groups, respectively.

### 4.3. Cell Culture

The human normal cervical epithelial cell line (HcerEpic) was kindly provided by Dr. Guoliang Huang from the Department of Biomedical Engineering, School of Medicine, Tsinghua University. The human cervical cancer cell line SiHa was kindly provided by Dr. Wei Han from the Institute of Health and Medical Technology, Hefei Institutes of Physical Science, Chinese Academy of Sciences. The human cervical cancer cell line CasKi was kindly provided by Dr. Tengchuan Jin from the Division of Life Sciences and Medicine, University of Science and Technology of China. The SiHa, CasKi and HcerEpic cell lines were cultured respectively in RPMI-1640 medium (Gibco, Waltham, MA, USA), DMEM medium (Gibco, Waltham, MA, USA), and a mixed medium (the volume ratio of F12/MEM medium to DMEM medium is 2:3), all supplemented with 10% fetal bovine serum (FBS) (ExcellBio, Shanghai, China) and 1% penicillin/streptomycin (Sparkjade, Jinan, China). The cells were incubated in an environment of 37 °C and 5% CO_2_.

### 4.4. Immunocytochemistry [44]

The HcerEpic cells were seeded onto 12-well plates with coverslips and cultured for 24 h, followed by treatment with 500 ng/mL LPS (L6529, Sigma-Aldrich, Saint Louis, MO, USA) for 1, 3, and 7 days. After washing the cells with phosphate-buffered saline (PBS), they were fixed with 4% paraformaldehyde (Biosharp, Beijing, China) for 20 min on slides. The cells were then permeabilized with 0.5% Triton X-100 for 30 min. To quench endogenous peroxidase activity, the cells were incubated with 3% hydrogen peroxide for 10 min and subsequently blocked with 10% goat serum (Solarbio, Beijing, China) for 45 min. The cells were then incubated overnight at 4 °C with primary antibodies against phospho-RELA (ser536) (sc-136548, Santa Cruz Biotechnolog, Santa Cruz, CA, USA), FN1 (WL00712a, Wanleibio, Shenyang, China), PLAU (WL02483, Wanleibio, China), ICAM1 (WL02268, Wanleibio, China), and Ki67 (WL1384a, Wanleibio, China). After washing with PBS, the cells were incubated with HRP-conjugated secondary antibody (KIT5020, Maixinbio, Fuzhou, China) for 30 min at room temperature. The cells were stained with DAB solution (DAB-0031, Maxinbio, China) for 2 min, followed by hematoxylin staining for 10–30 s. They were then immersed in 95% ethanol and absolute ethanol for 4 min each. The slides were sealed with neutral gum, observed under a microscope, and photographed. The percentage area (%Area) of positive staining for the target proteins was scored using ImageJ software (version 1.53e, USA).

### 4.5. Immunohistochemistry [44]

Tissue samples were fixed in 4% paraformaldehyde for 24 h, embedded in paraffin, sectioned into 2 μm slices, and baked at 60 °C for 4 h. The slices were dewaxed using xylene and rehydrated through a graded series of ethanol solutions. Antigen retrieval was performed by heating the slices in citrate buffer for 2 min, followed by cooling. The slices were then immersed in 3% hydrogen peroxide for 10 min, washed three times with PBS for 5 min each, and blocked with 10% goat serum for 45 min. After that, the slices were incubated with primary antibodies overnight at 4 °C. After rinsing three times with PBS, the slices were incubated with secondary antibodies for 30 min. Subsequently, the slices were incubated with DAB chromogen for 2 min, stained with hematoxylin, rinsed with water, dehydrated with ethanol, and sealed with neutral gum. The stained sections were observed under a microscope. ImageJ software was used to segment the positive areas for the target protein, and the percentage area of positive staining and the mean optical density (Mean OD) of the positive areas were calculated.

### 4.6. Western Blot [45]

The instructions for this step are as follows: Prepare cell lysis buffer using a ratio of RIPA buffer: protease inhibitor: 0.5M EDTA = 100:1:1 (Sparkjade, Jinan, China). Separate proteins using 10% SDS-PAGE (Sodium Dodecyl Sulfate Polyacrylamide Gel Electrophoresis). Transfer the proteins to a PVDF membrane (Immobilon-P, Millipore, Burlington, MA, USA) via immunoblotting. Incubate the membrane with 5% BSA (BioFroxx, Einhausen, Germany) at room temperature for 1 h, followed by overnight incubation with primary antibodies at 4 °C. The primary antibodies used include anti-RELA (sc-8008, Santa Cruz Biotechnolog, Santa Cruz, CA, USA), anti-phospho-RELA (ser536) (sc-136548, Santa Cruz Biotechnolog, USA), anti-N-Cadherin (380671, ZENBIO, Chengdu, China), anti-E-Cadherin (201283, ZENBIO, China), anti-PLAU (A04352-1, BOSTER, Wuhan, China), anti-FN1 (WL03677, Wanleibio, China), and anti-ICAM1 (WL02268, Wanleibio, China). The next day, incubate the membrane with horseradish peroxidase-conjugated anti-mouse or anti-rabbit secondary antibodies (ZENBIO, China) at room temperature for 1 h. After washing three times with TBST, develop the immunoblots using electrochemiluminescence reagents (NCM Biotech, Suzhou, China) to observe the immune reactive bands.

### 4.7. Real-Time Quantitative PCR [46]

The HcerEpic cells were seeded in 6-well plates and cultured for 24 h, followed by treatment with LPS (0 and 500 ng/mL) for 6 h. Total RNA was extracted from the cells using the TransZol Up Plus RNA Kit (TransGen, Beijing, China). The quality and concentration of the RNA were determined using a Nanodrop spectrophotometer (Implen GmbH, München, Germany). The RNA was reverse transcribed into cDNA using the Two Step RT&qPCR Kit (Seven, Beijing, China), and RT-qPCR amplification was performed on the X960 Real-Time PCR Detection System (Heal Force, Shanghai, China). β-Actin was used as the internal reference gene, and the expression level of the target gene was calculated using the 2^−ΔΔCt^ method. The primers used were synthesized by Sangon Technologies (Shanghai, China). Primer sequences for the target genes are listed in Supplemental Table S3.

### 4.8. Cell Viability Assay [47]

HcerEpic, SiHa, and Caski cells were seeded into 96-well plates at appropriate densities, with six replicate wells for each treatment condition. After 24 h, the cells were treated with LPS or SC75741 (TargetMol, Boston, MA, USA). After the appropriate treatment duration, Cell Counting Kit-8 (CCK-8) (TargetMol, MA, USA) was used to prepare a working solution by mixing CCK-8 solution with culture medium in a ratio of 1:10, and 110 μL of CCK-8 working solution was added to each well. After incubation at 37 °C for 1 h, the optical density at 450 nm was measured using a microplate reader.

### 4.9. Wound Healing Assay [48]

Horizontal and vertical lines were drawn on the bottom of 6-well plates using a marker pen to facilitate positioning during subsequent photography. The cells were seeded into the 6-well plates with lines on the bottom and cultured for 24 h. A vertical line was then scratched on the monolayer of cells using a 10 μL pipette tip, and the cells were washed with PBS. Serum-free culture medium containing LPS or SC75741 was added, and the cells were observed and photographed under a microscope at 0 h post-scratch. Thereafter, the cells were continuously cultured in a serum-free medium and were observed and photographed under a microscope every 12 h post-scratch for documentation.

### 4.10. Clone Formation Assay [49]

After counting, SiHa and Caski cells were seeded into 12-well plates at a density of 800 cells per well and cultured for 10–14 days. When visible small clones formed, the cells were washed twice with PBS and fixed with methanol at 37 °C for 30 min. They were then stained with 1% crystal violet for 30 min, and the excess dye was washed off with water. Finally, photographs were taken for documentation.

### 4.11. Statistical Analysis

All data were subjected to statistical analysis using SPSS (version 27.0), and statistical graphs were generated using GraphPad Prism (version 8.0.2). For comparison among multiple groups of quantitative data, one-way ANOVA was performed, followed by Tukey’s HSD test for multiple comparisons. For pairwise comparisons between groups, an independent sample t-test was used. All data were based on three independent experiments and expressed as mean ± SEM. *p* < 0.05 was considered statistically significant.

## Data Availability

The datasets presented in this study can be found in online repositories, as indicated in Section 4. All data related to this study are available from the corresponding author upon reasonable request.

## Supplementary Materials

The supporting information can be downloaded at: https://www.mdpi.com/article/10.3390/ijms26062493/s1.

## Abbreviations

CIN: Cervical intraepithelial neoplasia; CSCC: Cervical squamous cell carcinoma; HSIL: High-grade squamous intraepithelial lesion; HPV: Human papillomavirus; WHO: World health organization; EMT: Epithelial-mesenchymal transition; DEG: Differentially expressed genes; LPS: Lipopolysaccharide; PBS: Phosphate-buffered saline; GO: Gene Ontology; OS: Overall survival; pRb: Retinoblastoma protein; KM: Kaplan-Meier; TAMs: Tumor-associated macrophages.
