# Supplementary material for: NF-κB Activation Is Essential for Cervical Cell Proliferation and Malignant Transformation
Source: Int J Mol Sci. 2025 Mar 11;26(6):2493. doi: 10.3390/ijms26062493 (PMC11942554; doi:10.3390/ijms26062493)
Supplement: Supplementary file 1 [file ijms-26-02493-s001.zip › ijms-3468312-SI.pdf]

Supplemental Table S1. TRRUST predicts RELA as a key transcription factor regulating DEGs in CIN1, CIN3, HSIL, and CSCC groups

Lesion grade	Ranking of RELA	Numbers of DEGs regulated by RELA	p.value	FDR
CIN1	1	8	0.0080244	0.0028
CIN3	2	16	7.14×10^{-9}	2.55×10^{-7}
HSIL	2	40	2.39×10^{-7}	1.92×10^{-6}
CSCC	2	47	4.46×10^{-6}	5.58×10^{-5}

Table S1: Transcription factor predictions for differentially expressed genes across CIN1, CIN3, HSIL, and CSCC groups were performed using the TRRUST website. Transcription factors are ranked from highest to lowest based on the number of regulated differentially expressed genes.

Supplemental Table S2. DEGs Regulated by RELA in CIN1, CIN3, HSIL, and CSCC Groups

Lesion grade	Gene symbol
CIN1	*CCL20 EPCAM SAA1 COL1A2 CXCL12 ERAP2 NCAM1 LCN2*
CIN3	*CCL20 CFTR CXCL1 CXCL5 CXCL8 ELF3 EPCAM MMP9 PIGR PTGS2 SAA1 SERPINA3 TFF3 CXCL12 NCAM1 PTGFR*
HSIL	*ALOX5 CCR7 CFI CFTR CTSB CXCL1 CXCL5 EPCAM ERAP1 MMP14 MUC5AC PSMB10 S100A6 SAA1 SAA2 SOD2 TIMP1 TNFSF10 BHMT BMP2 CCL2 CCND1 CD69 CFLAR CXCR2 EDN1 F3 HBEGF HPSE HYAL1 IER3 IL18 IL1B IL1RN MYC PPARD PTGER4 PTHLH TRIB3 UPP1*
CSCC	*PLAU MMP1 MMP12 MMP13 ICAM1 FN1 SERPINE1 VEGFA CXCL1 BIRC3 BIRC5 CCL20 CCNB1 CXCL8 E2F1 EPCAM IL1A IGF2BP2 LIPE LTC4S PSMB10 STAT1 TNFSF10 AR BTG2 CCL2 CCND1 CCND2 CD69 CEACAM1 CXCL12 VMF CXCR2 EGR1 FAS HBEGF HMOX1 IL18 IL1RN F3 UPP1 MADCAM1 OLFM4 PTGER4 SERPINBB1 TFF3 CYP3A4*

Table S2: Based on the predictive analysis from the TRRUST website, RELA regulates 8, 16, 40, and 47 downstream DEGs in CIN1, CIN3, HSIL, and CSCC groups, respectively. Table S2 presents the names of these DEGs.

Supplemental Table S3. List of primers used in quantitative RT-PCR.

Gene symbol	GeneBank accession	Forward primer sequence (5'→3')	length	Tm	Reverse primer sequence (5'→3')	length	Tm	Product length
RELA	NM_ 001404 657.1	AGAGCAGCG TGGGGACTA	18	59.96	ATGGGATGAG AAAGGACAGG	20	56.59	111
Actin	NM_ 001101.5	TGTATGCCTC TGGTCGTACC	20	58.89	CAGGTCCAG ACGCAGGAT	18	58.70	116
